# PD62 Economic Evaluation Of Breast Cancer Genomic Testing For Risk Stratification In Brazil’s Private Healthcare Sector

**DOI:** 10.1017/S0266462325101943

**Published:** 2025-12-29

**Authors:** Ivan Zimmermann, Márcia Gisele Santos, Carlos Magliano

## Abstract

**Introduction:**

Breast cancer (BC) confers a high burden on the Brazilian population. Moreover, the possible overuse of adjuvant chemotherapy in patients with a low risk of tumor recurrence may subject these individuals to harmful side effects, without offering substantial therapeutic advantages. The implementation of genomic testing for risk stratification represents a promising strategy to optimize patient care.

**Methods:**

We assessed the economic implications of integrating the Oncotype DX Breast Recurrence Score Test® for stratifying BC patients according to their recurrence score (RS). A decision tree and Markov model estimated the long-term costs and outcomes linked to transitions between the health states of recurrence-free, distant recurrence, acute myeloid leukemia, and death. Patient distributions and probabilities of distant recurrence were obtained from the TAILORx (N0) and RxPONDER (N1) clinical trials. Local evidence on utility and overall survival was also considered. The analysis was carried out from the perspective of the Brazilian private healthcare system. Deterministic and sensitivity analyses were conducted.

**Results:**

Compared with clinical and pathological risk alone, adding the Oncotype DX test for all BC patients (N0 or N1) generated more quality-adjusted life years (QALYs) at lower costs (0.15 QALYs and −USD41,251.80). The three most efficient indications included: restricted to N1 (0.28 QALYs and −USD6,032.65); N0 restricted to high risk and N1 restricted to post-menopause (0.22 QALYs and −USD6,276.73); and N0 restricted to high risk (0.20 QALYs and −USD6.335,99). The main impact drivers were chemotherapy costs, chemotherapy prescription probabilities, and acute myeloid leukemia incidence rate. The sensitivity analysis indicated a high probability of the test being cost effective.

**Conclusions:**

The Oncotype DX test has a high likelihood of being a cost-effective strategy from the Brazilian private healthcare perspective. Alternative scenarios and test indications did not alter these conclusions.

